# Termite-Induced Injuries to Maize and Baby Corn under Organic and Conventional Farming Systems in the Central Highlands of Kenya

**DOI:** 10.3390/insects10100367

**Published:** 2019-10-22

**Authors:** John J. Anyango, David Bautze, Komi K. M. Fiaboe, Zipporah O. Lagat, Anne W. Muriuki, Sibylle Stöckli, Gladys K. Onyambu, Martha W. Musyoka, Edward N. Karanja, Noah Adamtey

**Affiliations:** 1 International Centre of Insect Physiology and Ecology (icipe), Nairobi P.O. Box 30772-00100, Kenya; anyangojohn@yahoo.com (J.J.A.); mmusyoka@icipe.org (M.W.M.); ekaranja@icipe.org (E.N.K.); 2 Kenya Agricultural and Livestock Research Organization (KALRO), Nairobi P.O. Box 57811-00200, Kenya; muriukianne@gmail.com; 3 Research Institute of Organic Agriculture (FiBL), P.O. Box 219, 5070 Frick, Switzerland; sibylle.stoeckli@fibl.org (S.S.); noah.adamtey@fibl.org (N.A.); 4 International Institute of Tropical Agriculture (IITA), B.P. 2008 (Messa), Nkolbisson, Yaoundé, Cameroon; K.Fiaboe@cgiar.org; 5 Zoology Department, Jomo Kenyatta University of Agriculture and Technology, Nairobi P.O. Box 62000-00200, Kenya; zbisieri@yahoo.com (Z.O.L.); glaonya@yahoo.com (G.K.O.)

**Keywords:** termites, maize damage, farming systems research, organic farming

## Abstract

Termite-induced injuries to maize and baby corn were evaluated in on-going comparison experiments on organic and conventional farming systems at two trial sites in the Central Highlands of Kenya (Chuka and Thika). The farming systems were established in 2007 at two input levels: Low input level, representing subsistence farming (Conv-Low, Org-Low) and high input level, representing commercial farming (Conv-High, Org-High). Termite-induced injuries to maize and baby corn, such as tunneling the stem or lodging the whole plant were assessed over two cropping seasons. The lodging occurred exclusively at Thika. It first became apparent in the Org-Low system, with most of lodging occurring during the vegetative stage. Baby corn grown under high input systems showed increasing lodging from the late vegetative crop stage and peaked before the final harvest. Tunneling was recorded at both sites, but was generally below 5%, with no significant differences between the farming systems. Overall, the injury patterns caused by termites appear to be a function of the plant growth stage, termite colony activities, trial site, and the types and levels of fertilizer input. Thus, the management practice used in each farming system (organic or conventional) might have greater influence on crop injuries than the type of farming system itself or the termite abundance within each system.

## 1. Introduction

The comparison experiments to generate field data on organic and conventional farming systems were initiated in 2007 in Kenya by the Research Institute of Organic Agriculture (FiBL) and their local partners, the Institute of Insect Ecology and Physiology (icipe) and the Kenyan Agricultural and Livestock Research Organization (KALRO). The study aims to compare the performance of organic and conventional farming systems in the tropics on productivity, profitability and sustainability. However, in the trial plots, the presence of termites, as major macrofauna, was reported repeatedly and confirmed by a first study [1].

Termites (Isoptera) are a large and diverse group of insects, globally consisting of over 2800 species in about 300 genera. Africa has greater termite diversity than any other continent [2]. Within the literature, there is a distinct dichotomy between termites being depicted as ‘pests’ attacking structural timber, rangelands, crops and trees, and the ecological literature which argues that they provide several ecosystem services [3]. Jouquet et al. [4] and Kahn et al. [5] summarize the services provided by termites as including: (i) contributing to the consumption and mineralization of plant and animal materials; (ii) increasing the diversity of vegetation, animals, and microbial communities; and (iii) altering the chemical and physical properties of soils. The effect on soils is mentioned by several other authors. Khan et al. emphasize the role of termites as soil engineers and processors, especially given that they are frequently very abundant, represent a significant biomass, and construct extensive networks of galleries and nests, therefore, they have a significant impact on pedogenesis, soil properties and functions [6]. Soils populated by termites are better drained, more stable and have higher levels of soil organic matter. In addition to termites’ impact on ecosystems, they have also been used in scientific research, medicine, and technology (e.g., bionics, geology) [7].

Despite their ecological role, termites can become a problematic issue for agriculture, forestry, and construction. In agriculture, termites attack a variety of plants from the seed to the mature plant as well as during storage [8]. Seedlings are either cut just below or above the soil surface, and usually, they are entirely destroyed. Injuries to maturing plants happen either directly, when termites enter and consume the crop roots, lodging the whole plant or indirectly, as injured plants become more susceptible to pathogens. Damage to stored products generally results in invasion by secondary pests (such as fungi). Crop yield losses are higher in rain-fed crops during dry periods, in lowland areas, in plants under stress, and non-indigenous plants [7]. The crop yield losses caused by termites can reach up to 100% in some cases in eastern Africa [9], but a study from Western Kenya shows the majority of losses in maize is between 11 and 20% [10]. In Africa, there are approximately 20–50 termite species that can be considered as pests and cause economic damage to groundnuts, maize, sugarcane, yam, cassava, and cotton. However, maize is considered as the most often damaged crop by termites among all cereal crops [8]. Important species considered as crop pests are in the subfamily Macrotermitinae (*Macrotermes*, *Odontotermes*, *Pseudacanthotermes*, *Ancistrotermes* and *Microtermes*) [8].

Nonetheless, termites do not become pests by themselves. Often, agricultural practices such as deforestation, overgrazing, ecosystem simplification or hunting lead to termites acquiring pest status [11]. In environments unaffected by humans, termites have little or no negative impact [8]. Furthermore, some authors argue that termites might play an essential role in future agricultural scenarios where external inputs to agricultural ecosystems become uneconomic and unsustainable, and the termites’ ecosystem services could be a feasible alternative [4]. Bignell [6] suggests that termite conservation needs to be linked to soil fertility and sustainable subsistence agriculture to counteract rapid land-use changes and increasingly disturbed or reduced natural habitats. However, there is little knowledge about how termite abundance, diversity, and damage are affected by farming systems. Studies from Nigeria showed that termites are sensitive to land-use changes: species richness decreases with land-use intensification, while encounters, party size, and activity increases [12].

These considerations led us to include a study on termite pest activities within the long-term experiments (LTE) in Kenya. The objectives of the studies were to compare organic and conventional farming systems and more, specifically: (i) to determine the incidence of different injuries (lodging and tunneling) at different crop stages; and (ii) to determine the termite species causing the injuries. The study hypothesis was that termite pest status is influenced by farming systems, and as termites were observed to be more abundant in the organic systems with high inputs of fertilizer and irrigation (see [1]), a greater incidence of injuries was expected in this farming system than in the others.

## 2. Materials and Methods

### 2.1. Study Area and Experimental Design

The field experiments on termite pest activities were carried out in 2014 and 2015 at two trial sites, Thika and Chuka, in the Central Highlands of Kenya (Table 1 and [13] for detailed information).

At each site, conventional (Conv) and organic (Org) farming systems are being compared at two levels of input: high input (High) using the recommended rates of fertilizer and pesticides as well as supplementary irrigation (which represents commercial large-scale production) and, low input (Low) using limited amounts of fertilizer and pesticides (representing smallholder production, mainly for subsistence). The management practices of these four farming systems are being applied on experimental plots of 8 × 8 m (64 m^2^; net plot 6 × 6 m) arranged in a Randomized Complete Block Design (RCBD), replicated four times at Chuka and five times at Thika. The crop rotation consists of maize, beans, leafy vegetables, and potatoes, which are planted in a 3-year-6-season crop rotation with a long and a short season. We investigated the effects of termites on maize (*Zea mays*)—either baby corn (var. Pannar 14) or grain maize (var. H513) during the first growing seasons of 2014 and 2015. Baby corn was planted as the sole crop in the high input level (organic and conventional) systems, whereas in the low input (organic and conventional) farming systems, grain maize was intercropped with common beans (*Phaseolus vulgaris* var. GLP 92). The two maize cultivars are grown for different purposes: baby corn, for the young ears which are consumed as a vegetable, and the grain maize for flour production. Baby corn is commercially grown as a sole crop for export and urban domestic markets. It was planted at a spacing of 75 cm between rows and 30 cm between plants with one baby corn seed per hole. The grain maize was planted as an intercrop of one row of maize alternated with a row of common beans, spaced at 75 cm between rows and 60 cm between maize and 30 cm between bean plants (two seeds per hole).

The maize was planted after the land was prepared with hand hoes. The plots were kept weed-free by manual weeding. Sowing was done within the same week at the two sites. The Conv-Low system received fresh farm-yard manure and inorganic mineral fertilizers: Triple-Super-Phosphate (TSP), Di-Ammonium-Phosphate (DAP), and Calcium-Ammonium-Nitrate (CAN) applied at an average rate of 31 kg N and 18 kg P ha^−1^. The Conv-High system was supplied with decomposed farm-yard manure and inorganic fertilizers TSP, DAP, and CAN at the rate of 113 kg N and 60 kg P ha^−1^. The Org-Low system was supplied with decomposed farm-yard manure and phosphate rock (PR) at the same N and P rate as Conv-Low. The Org-High system was supplied with compost, liquid manure from *Tithonia diversifolia*, and various crop residues as mulch (maize stover, dry grass, and *Tithonia*) at the same N and P rate as Conv-High. In addition, a relay legume crop (*Mucuna pruriens*) was planted in Org-High 40 days after planting the maize. The high input systems received supplementary water through drip irrigation. The pest and disease management and especially the termite control methods were different depending on site and system. At Chuka, no termiticide was used in both seasons. The pesticides used in the system were mainly to control stemborer—Bulldock (Pyrethroid) in Conv-High, Halt (*Bacillus thuringiensis*) in Org-High and wood ash in low input systems. At Thika, we used the termiticides Dragnet (Permethrin; ~20 mL in 5 L; applied once) and Concord (Cypermethrin; ~4 mL in 2 L water; applied twice) in the conventional systems in 2014 and 2015, respectively. In the organic systems, we used icipe formulation no. 30 (fungus *Metarrhizium anisopliae*) with different carrier materials (liquid: corn oil; solid: rice). The formulation was used once in 2014 (2 kg solid carrier) and twice in 2015 (1.7 kg solid carrier as well as 4 and 5 mL liquid carrier in Org-Low and Org-High, respectively). In addition, the same pesticides for stemborer control at Chuka were used at Thika.

### 2.2. Data Collection

**Figure 1:**
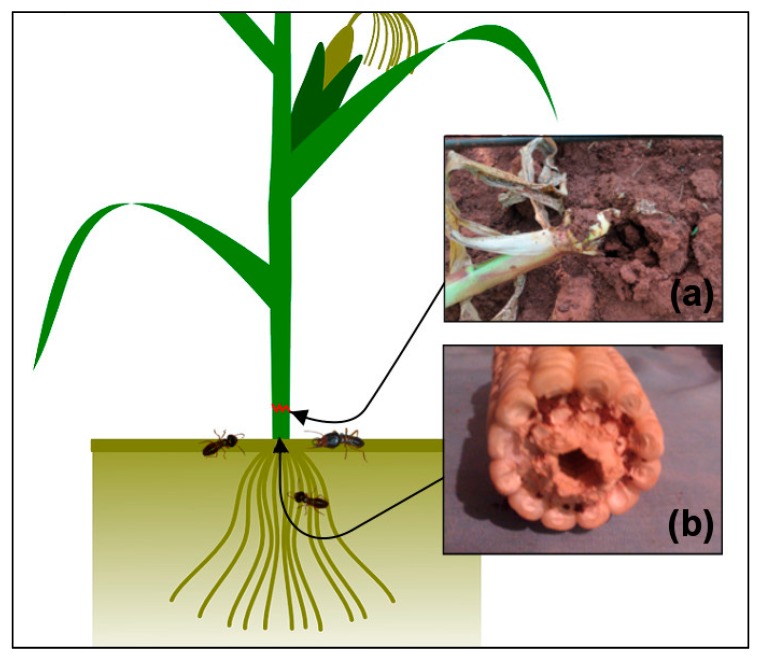
Lodging (**a**) and tunneling (**b**) done by termites on maize plants.

We have measured two types of injuries in our experiment, lodging and tunneling (Figure 1). The lodging was often recorded on previously healthy-looking plants that were suddenly cut by termites, then fell and became lodged into galleries. The lodging often occurred at the soil surface (the base of the stem). The termites causing the lodging often attacked the plants overnight or early morning in a group. These lodged mature plants were then exposed to other secondary pests, including rodents and fungi. Tunneling happens when termites enter the plant roots and extend their foraging by excavating the inside of the stem. Maturing and physiologically mature plants which are affected by tunneling can remain intact, although they have been hollowed from the inside and filled with a mixture of faeces and soil-particles. They can easily be toppled by the slightest movement. Plants affected in an early growth stage experience an interruption of their nutrient and water flow and therefore wilt.

Lodging injuries were sampled weekly from the 1st to the 20th week after emergence (WAE) of the crops, by counting the number of lodged plants within each plot at both sites and seasons. We expressed lodging as number of lodged plants relative to the total plant population of the plot (i.e., the % of affected plants). Tunneling of the stem was detected once by destructive sampling, conducted at the final harvesting at both sites. Twelve plants per plot were uprooted using a hand hoe and dissected. The plants showing perforations and with soil inside the plant were recorded. We expressed tunneling as the relative number of affected plants to sampled plants per plot (the % of affected plants).

A weekly sampling of termites was carried out every season from 1st to the 20th WAE in 4 quadrants within the net plot areas of the experimental plots. Termites were sampled from 20 × 10 × 10 cm monolith soil profiles in the topsoil (0–20 cm) and subsoil (20–40 cm). The sampled termite soldiers were identified in the field as much as possible through morphological assessments using a hand lens. The soldier genus identification was later confirmed at the National Museum of Kenya, Nairobi, using the standard determination keys [17,18]. The detailed results can be found in [1].

### 2.3. Data Analysis

The data obtained were subjected to statistical analysis using R-Statistical software [19]. Data on lodging was checked for outliers and for complete separation using the *detect_separation* function from the *brglm2* package [20]. Complete separation was found to be true. Thus, we used the *brglm* function from the *brglm* package to conduct a bias-reduced generalised linear model [20] with a binomial distribution using farming system, sampling date, and year (and their interactions) as factors. Afterwards, we performed (i) an analysis of deviance (Wald chi-square test) to check the significance of each factor (using the *Anova* function from the *car* package [21]), and (ii) a *post-hoc* Tukey test for pairwise comparison of farming systems (using the *emmeans* function from the *emmeans* package [22]). Data on tunneling were tested with a generalized linear mixed-effect model with a binomial distribution using the farming system, season, and site as fixed factors, and the block as a random factor. We used the *lmer* function from the *lme4* package [23] to set up the model. An analysis of deviance and a *post hoc* test were also performed in the same way for lodging, where relevant. Assumptions for each model (heteroscedasticity and distribution) were graphically tested. The significance level for all statistical tests was set at *p* < 0.05.

## 3. Results

**Figure 2:**
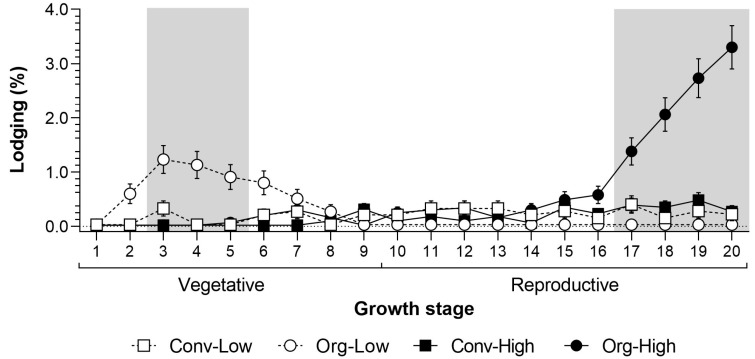
Lodging (% affected plants per plot and growth stage) in the experiment at Thika in the Central Highlands of Kenya. Grey areas show periods when there was a significant difference between the farming systems.

Lodging occurred exclusively at Thika. Therefore, we only tested for significant differences over the seasons and systems at this trial site. The analyses showed a significant effect for farming system and sampling date (Table 2). The first signs of lodging were recorded in the organic low input system at 2 weeks after emergence (WAE; Figure 2). The maximum lodging under Org-Low occurred at between 3 and 5 WAE and was significantly greater than in all the other farming systems. However, the lodging of young maize plants could be compensated for through replanting. Lodging in the later vegetative or the reproductive stages was economically more severe. During these later stages lodging was similar in both low-input systems.

Baby corn grown under organic high input systems showed more severe lodging in the late reproductive crop stage, which increased gradually and peaked at 20 WAE. At this stage (17 to 20 WAE), lodging was significantly higher under Org-High than in all the other farming systems. However, at this time the crop was becoming senescent, producing a minimal amount of cobs. During peak harvesting from 10 to 17 WAE, lodging was minimal. Field observations and subsequent identification of termites showed that lodging was closely associated with the following species: *Odontotermes somaliensis*, *Macrotermes herus*, *M. michaelseni*, *Pseudacanthotermes spiniger* and *P. militaris*.

Tunneling occurred at both sites and in both seasons. The statistical analysis of the tunneling showed no significant farming system effects or significant interaction with farming systems (Table 2). Tunneling was generally below 5% in all farming systems showing average values of 2.3%, 2.8%, 1.4%, and 4.2% for Conv-Low, Org-Low, Conv-High and Org-High. In general, tunneling can be classified as minor, because the affected plants often remained intact, retaining their cobs through to harvest. Field observations revealed that the termite genera most commonly associated with tunneling symptoms were *Microtermes*, *Amitermes* and *Ancistrotermes*.

## 4. Discussion

Anyango et al. [1] could show that the termite abundance and diversity were generally higher in Org-High at both experimental sites (Chuka and Thika) and in both maize seasons (2014 and 2015). Average termite abundance and diversity were lower in all the other farming systems. Thus, it was expected that termite-induced injuries during the same time period and at the same experimental sites would be significantly higher in Org-High throughout the season. However, the results on maize injuries presented in this research indicate that the presence of termites is not automatically linked to lodging and tunneling in either variety of maize. Even though few termites were present in Org-Low, this farming system showed the highest rate of lodging in the early vegetative crop stage. By contrast, Org-High plots showed minimal injuries during the vegetative stage despite the higher presence of termites. In addition, lodging only occurred at Thika, and no lodging occurred in any of the farming systems at Chuka, despite this site having a higher abundance of termites. These variations might be attributed to several reasons, discussed below.

Firstly, differences in fertilizer input and the induced plant health status could have led to differences in lodging during the initial stages at Thika. The Org-Low system received low amounts of decomposed farm-yard manure and phosphate rock, which only slowly release nutrients to the plants. This led the seedlings to display stunted growth and be less vigorous than those in the other systems, due to a nutrient deficiency. This might have been attracting the termites to attack and damage the plants, despite their low abundance. The lodging stopped when the plants became greener and started growing more vigorously (from 7 WAE onwards). The maize plants then became less woody and maybe less attractive, resulting in less lodging through till the harvest (as also described by [24,25]). This finding is in contrast to [26], in which it is stated that termites only cause losses in mature plants because the young maize seedlings repel termites through the phenols and cyanide compounds they produce. It would seem that this is only valid for plants which do not suffer nutrient or water deficiencies. Plants in the conventional systems, which received readily available nutrients from inorganic fertilizer and farm-yard manure, as well as Org-High plots, which received a high level of organic fertilizer inputs (compost, mulch + irrigation) showed more vigor in their growth, with minimal injuries during the vegetative crop stage. We hypothesize that the inputs boosted the health of the plants and made them less attractive to being attacked by termites in the early stages, even where there was a high presence of termites (Org-High). These results emphasize that termite attacks on maize plants depend on the health status of the crop as opposed to its growth stage. This is in contrast to findings by [27,28,29] who state that termites only cause damage to mature maize.

The availability of organic plant material can also influence termite behavior. A considerable amount of organic material was applied to the Org-High system throughout the season, which most probably led to an increase in termite abundance, as these materials created a suitable habitat and also provided a food source for the termites. However, it is not clear if the addition of these materials also decreased crop injuries by offering an alternative food source for the termites. Other authors have shown that the presence of organic material increases termite abundance and activity, but that termite damage was sometimes not affected [30], or if it was affected, did not lead to significant yield losses [31]. Nonetheless, the organic material did not prevent injuries towards the end of the reproductive stage in the Org-High plots at Thika. This might be attributed to the higher lignin and cellulose content in the plants of the Org-High system at later crop stages (in this study the proportions were 4% and 23% respectively at Chuka, and 9.25% and 33.25% at Thika—data not shown) and the diversion of the applied organic material. Higher lignin and cellulose content resulting in crop lodging, has also been noted in other studies by [32].

Rainfall patterns are another factor that affects termite behavior and crop injuries. Heavy rainfall generally inhibits termites’ foraging activities, as it limits their ability to construct foraging tunnels, or they quickly drown. Thus, the termites stay in their nests during the rainy period, running down the colony’s food reserve and only start to forage again when it starts raining less (as observed by [29]). In addition, when it is raining less, the crops are more likely to face some moisture stress, and the vegetation cover decreases, thus coinciding with crops more susceptible to termite-induced injuries again (as noted by [29,33]). This would apply to all farming systems and does not explain the reported differences, but it might be a reason for the differences between the two sites (see Table 1). Termites were twice as abundant at Chuka as Thika, but there was a similar amount of tunneling and no lodging). Chuka is more prone to heavy rainfall and flooding than Thika (own data, see [13]), which would shift termite activity to deeper soil layers and prevent surface foraging. This could also explain the higher numbers of termites found in the subsoil (20–40 cm depth) at Chuka than in the substrate layer [1]. Ref. [34] also showed that flooding prevents termites from foraging freely. In addition, the lower rainfall at Thika would most likely have affected the availability of other food sources in the environment, leading termite colonies to attack and forage on crops grown in the plots for food in the dry spells. This is also confirmed by our field observation showing termite aggressively felling maize plants at Thika, usually overnight or early in the morning, after which they returned to their nests within the plots or the surrounding areas.

Lenz et al. [8] describe that the type of injuries is also termite species-specific. In our experiment, we found a total of 9 termite genera, mainly from three sub-families: (i) Macrotermitinae (ii) Termitinae and (iii) Nasutitiermitinae. However, only three out of the nine species found (*Odontotermes*, *Macrotermes*, and *Pseudacanthotermes*) were closely associated with lodging. Other authors have also identified these species as being responsible for attacking maize crops [8,29,35,36]. However, the scientific literature describes the severity of termite attacks differently, depending on the species, crop, and climatic region [37].

In the conventional systems, where termite abundance was generally low, lodging was also low (<1%). The low abundance might be attributed to the chemical pesticides applied, which controlled termites effectively. Other authors have found that the efficiency of chemical pesticides can vary. Nyagumbo et al. [30] tested different chemical pesticides, botanicals (e.g., *Lantana camara*), and mineral substances (wood ash, lime) for termite control, but only the chemical pesticide (active ingredient fipronil) proved effective in reducing crop lodging. Ref. [35] reported that removal of the queen and mound firing/smoking was the most effective method used by farmers for controlling some termite species. In the current trial, the effectiveness of the chemical pesticides was variable, being efficacious over the vegetative and early maturity stages of the maize crop but less effective later.

Tunneling could not be studied in detail in this study because the destructive sampling method only allowed for sampling at the end of the growing season. Our results show that tunneling only occurred occasionally and the maize crop was able to carry the cobs to the final harvest stage, but we do not know how much it affected the crops’ development. Overall, tunneling only had a minor detrimental effect in the experiment. The termite genera most commonly associated with tunneling were *Microtermes*, *Amitermes*, and *Ancistrotermes*. The likely reason for them injuring maize in this way may be related to their smaller sizes, their preferred habitat, i.e., the lower soil surface and their ability to penetrate root hairs and plant tissues [38].

We did not make a detailed study of termite-induced yield losses in this experiment, because the experimental setup does not allow us to draw a direct conclusion from injuries to crops yields. The farming systems differ in several management practices (e.g., amount and quality of nutrients supplied, amount of irrigation water, type of pesticides, and planted crops). Therefore, the crop yield is a response to several input factors also, including termite injuries (and positive effects). However, we assume that the yield of grain maize in Org-Low was not necessarily affected by the lodging, because the lodged plants during the early vegetative phase were compensated through replanting. The yield of grain maize was higher on Org-Low in 2014 and 2015 (~2.0 and 4.5 Mg ha^−1^) than in Conv-Low (~1.5 and 4.0 Mg ha^−1^) at Thika, showing that termite lodging presented no distinct disadvantage for the Org-Low system. By contrast, yields for Org-Low at Chuka in both seasons (3.0 and 4.5 Mg ha^−1^) were less than in Conv-Low (3.5 and 5.0 Mg ha^−1^), even though there was no termite lodging in Org-Low. We cannot determine if termite lodging in Org-High caused significant losses, even though yields in Org-High were lower than or similar to those in Conv-High (in both seasons and sites). We assume that the yield losses were low because most of the severe lodging in Org-High appeared after the peak harvest of baby corn.

## 5. Conclusions

Termite-induced injuries in farming systems can be influenced by several management practices associated with different farming systems. The organic high input system had a higher abundance of termites as it provided them with more food sources and preferable conditions. However, this did not necessarily lead to greater injuries to the maize crop or yield loss. This suggests that termites becoming a ‘pest’ is dependent on other factors such as ecosystem deterioration. Termite attacks on crops and trees can be reduced to an acceptable threshold level by improving the surrounding ecosystems and as [4] state, encouraging predators and entomopathogens of termites (such as fungi, ants, spiders, beetles, and lizards), and providing other food sources. Termites are not necessarily harmful to farming systems and fulfill quite important ecological functions that support the sustainability of farming systems.

## Figures and Tables

**Table 1:** The site characteristics of the farming systems comparison trial sites at Chuka and Thika, Central Highlands of Kenya.

Parameter	Unit	Chuka	Thika
GPS		37°38.792′ N 0°20.864′ S	37°04.747′ N 1°0.231′ S
Altitude	m a.s.l.	1458	1500
Mean Annual Temperature	°C	19.2–20.6	19.5–20.7
Mean Annual Rainfall	mm	1373 (bimodal)	840 (bimodal)
Agroecological Zone		UM 2 (Main Coffee Zone)	UM 3 (Sunflower-Maize Zone)
Soil classification ^1^		Humic Nitisols	Rhodic Nitisols

^1^ based on FAO world reference base for soil resources ([14,15,16]); m a.s.l., meters above sea level.

**Table 2:** Results of the Wald-χ^2^-Test for the fixed effects on lodging and tunneling (% affected plants) in the farming systems comparison trial sites at Chuka and Thika.

Factor/Interaction	df	Lodging		Tunneling	
		χ^2^	*p* > χ^2^	χ^2^	*p* > χ^2^
Farming systems	3	127.3752	<0.001	2.9524	0.3990
Site	1	n.a.		0.2495	0.6175
Season	1	0.3607	0.5781	0.0380	0.8455
Sampling date	19	236.3193	<0.001	n.a.	
Farming system x site	3	n.a.		0.4794	0.9234
Farming system x season	3	4.7070	0.1946	0.6050	0.8953
Farming systems x date	57	193.4888	<0.001	n.a.	

df, degrees of freedom; n.a., not applicable.
